# Editorial: Emerging heterocycles as bioactive compounds

**DOI:** 10.3389/fchem.2023.1202192

**Published:** 2023-04-26

**Authors:** Giovanna Li Petri, Ralph Holl, Virginia Spanò, Marilia Barreca, Ignazio Sardo, Maria Valeria Raimondi

**Affiliations:** ^1^ Department of Biological, Chemical and Pharmaceutical Sciences and Technologies (STEBICEF), University of Palermo, Palermo, Italy; ^2^ Drug Discovery Unit, Ri.MED Foundation, Palermo, Italy; ^3^ Institute of Organic Chemistry, University of Hamburg, Hamburg, Germany; ^4^ German Center for Infection Research (DZIF), Partner Site Hamburg-Lübeck-Borstel-Riems, Hamburg, Germany

**Keywords:** heterocycles, new chemical entities, natural products, purine nucleoside phosphorylase, pantetheinase sulfhydrylase activity, quinazoline

The design and development of a drug is a very long process that generally takes many years of research. The laborious and expensive drug development pipeline, still characterized by a low success rate, requires several steps, including target identification, hit generation, hit-to-lead optimization, and preclinical/clinical evaluation. However, assessment of preclinical safety and potential efficacy in clinical trials are the Achilles’ heels of the study, as the most candidates halt their race to market due to pharmacokinetic (PK) issues. The hit-to-lead optimization, which represents a crucial step in the early drug discovery process, aims at improving the drug-likeness by modifying physicochemical properties, improving pharmacokinetic/pharmacodynamic (PD) profiles, and reducing off-target activities. Increasingly, lead optimization may benefit from the support of *in silico* studies for target identification and validation, as well as for Quantitative Estimation of Drug-Likeness (QED) predictions (Bickerton et al., 2012) and the adsorption, distribution, metabolism, excretion, and toxicity (ADMET) score (Guan et al., 2019) by adopting machine learning algorithms based on different molecular descriptors which allow to discriminate drug-like and non-drug-like compounds with high accuracy of 90%. Artificial Intelligence and -*omic* sciences can speed up the drug discovery process, but they should be combined with innovation, effectiveness, and efficiency for the construction of new organic molecules, always trying to follow the twelve principles of Green Chemistry (Anastas and Eghbali, 2010). However, daily, academia and large pharmaceutical companies have to deal with two major problems in their laboratory routines: high quantities of hazardous chemical waste in the production and purification processes and long reaction times. The environmentally friendly synthetic approaches, including mechanosynthesis, sonosynthesis, electrosynthesis, and microwave assisted organic synthesis (MAOS), and combinatorial and automated synthetic methods allow to overcome these issues, enabling greener chemistry in a shorter timescale. For example, MAOS in the synthesis of organic compounds, polymers, inorganic materials, and nanomaterials allows to obtain the desired molecules selectively, in higher yields, and in a few minutes compared to conventional reflux heating, by monitoring parameters like temperature, pressure, and ramping of temperature, and choosing not flammable or corrosive and non-volatile media solvents (Raimondi et al., 2019).

Heteroatomic fragments and heterocyclic scaffolds are very common in molecules with therapeutic properties because they aid to modify physicochemical properties and accomplish the best ADME/Tox results for drug candidates. In fact, heterocycles are usually used to optimize potency and selectivity through bioisosteric substitution of a variety of functional groups (Raimondi et al., 2012; Cascioferro et al., 2016; Barreca et al., 2021; Cruz, 2022). Moreover, more than 75% of the heterocyclic derivatives currently in clinical use contain at least two heteroatoms (Maggio et al., 2012; Sharma et al., 2020). According to a recent analysis conducted by de la Torre and Albericio (2022), in 2021, among the 36 new chemical entities (NCEs) approved by the US Food and Drug Administration (FDA), almost 50% are aromatic nitrogen heterocycles; and, from April 2020 to February 2022, triazole, tetrazoles, imidazoles/benzimidazoles, pyrimidines, and quinolines were the most frequently used building blocks in medicinal chemistry programs (Ebenezer et al., 2022). Also, statistical data reveals that more than 85% of bioactive compounds contain at least one nitrogen atom in their structure (Heravi and Zadsirjan, 2020; Barreca et al., 2022). Natural plant-, microorganism-, and animal-based biomolecules represent an important source of inspiration in the 21st century. About a quarter of all FDA-approved drugs are plant based, e.g., the strong opiate morphine mostly used for pain medication. Whilst about a third of FDA-approved drugs over the past 20 years are based on natural products or their derivatives, including antibiotics such as tetracycline from *Streptomyces aureofaciens*, artemisinin from *Artemisia afra*, doxorubicin from *Streptomyces peucetius*, cyclosporine from *Tolypocladium inflatum*, and so on (Thomford et al., 2018; Raimondi et al., 2020).

The Research Topic “Emerging Heterocycles as Bioactive Compounds” encompasses a Research Topic of one review and three original research articles focusing on the synthesis and biological evaluation of novel bioactive compounds.

Inspired by the biological properties of natural products (NPs), Huang et al., report the design, synthesis and the biological evaluation of a series of natural product-derived compounds which integrate three different moieties: *L*-menthone, pyrimidine, and urea. *L*-Menthone is one of the main components of the essential oil of *Mentha Canadensis* L. and can be prepared by oxidation of menthol. On the other hands, pyrimidine and urea compounds have received considerable attention in synthetic chemistry due to their diverse bioactivities. The new compounds have the potential to alter the overactivation of PI3K/Akt/mTOR cell grown signalling pathway in cancer. The *in vitro* antitumor activity was investigated against four cancer cell lines, including Hela, MGC-803, MCF-7, and A549. Cell cycle, cell apoptosis and western blot analyses confirmed the mechanism of action predicted by network pharmacology analysis and molecular docking.

The second article by Khandazhinskaya et al. describes the synthesis of a new series of 5′-norcarbocyclic fleximers bearing an artificial sugar fragment with a pyrazole-containing heterocyclic base, and the inhibitory properties in respect to purine nucleoside phosphorylase (PNP). Today, fleximer analogues of nucleosides are in the crossroad of heterocyclic and nucleoside chemistry, and this class of compounds is very promising because of a wide spectrum of biological activities. Specifically, the design with the fleximer aza/deazapurine bases can ensure additional conformational freedom in order to maximize structural interactions in the active site of the target enzyme while maintaining the structural similarity with a normal nucleoside substrate. Thus, helping with better understanding of enzyme specificity. The new compounds interact with the active site of the PNP of *E. coli* like natural heterocyclic bases, showing weak, non-competitive inhibitory activity.

Pantetheinase is an enzyme that in humans is encoded by the *VNN1* gene. The main effect of vanin-1/VNN1 is related to its pantetheinase sulfhydrylase activity, which can hydrolyze pantetheine into pantothenic acid and cysteamine. The enzymatic activity of vanin-1/VNN1 has been found to be essential in the development of many diseases. Wang et al. used fluorescent and bioluminescent probes to establish a series of visual screening methods and evaluate the inhibitory activity of a number of novel VNN1 inhibitors with different backbones. The authors demonstrated that VNN1 has a role of a pro-inflammatory agent during the onset of inflammatory bowel diseases (IBDs). Thus, the development of VNN1 inhibitors can provide a new avenue for effective IBD treatment to alleviate suffering.

Finally, the review article by Tamatam et al. describes methodologies and mechanistic insights for the synthesis of nitrogen-containing heterocyclic quinazolines via transition metal-catalysed reactions, paying attention to the advantages, limitations, and future perspectives of quinazoline synthesis via such reactions.

In conclusion, this Research Topic of articles demonstrates the importance of the heterocycles of natural or synthetic origin in molecules with therapeutic properties and recent advances in the preparation of the quinazoline scaffold.
